# Salvage Single-Port Transvesical Robotic Radical Prostatectomy Following High-Intensity Focused Ultrasound (HIFU) Therapy

**DOI:** 10.1590/S1677-5538.IBJU.2025.0744

**Published:** 2026-02-02

**Authors:** Mohamad Watfa, Nicolas A. Soputro, Abdulrahman Al-Bayati, Karim Daher, Salim Younis, Samarpit Rai, Rui M. Bernardino, Lin Wang, Zeyad R. Schwen, Ruben Olivares, Riccardo Autorino, Jihad Kaouk

**Affiliations:** 1 Glickman Urological Institute, Cleveland Clinic Ohio USA Glickman Urological Institute, Cleveland Clinic, Ohio, USA

## Abstract

**Introduction:**

Focal therapy with high-intensity focused ultrasound (HIFU) has emerged as a treatment option for selected patients with localized prostate cancer; however, disease recurrence requiring salvage intervention remains a recognized challenge (1–5). Salvage radical prostatectomy is technically demanding due to post-ablative tissue changes, which may compromise oncologic and functional outcomes (6,7). Herein, we describe the surgical technique and clinical outcomes of salvage robotic-assisted radical prostatectomy (RARP) performed using a single-port (SP) transvesical approach following HIFU.

**Materials and Methods:**

The index case was a 57-year-old man with a history of right hemigland HIFU for ISUP Grade Group 2 prostate cancer. During routine surveillance four years after HIFU, his PSA rose to 3.64 ng/mL, prompting repeat biopsy. Biopsy confirmed clinically significant recurrent prostate cancer both within and outside the prior treatment field, with bilateral involvement. Preoperatively, the patient reported satisfactory erectile function, with a Sexual Health Inventory for Men (SHIM) score of 25/25. After informed consent, salvage transvesical SP-RARP was performed. Dissection was carried out with anticipation of post-ablation tissue changes, and bilateral nerve-sparing was incorporated to optimize functional outcomes.

**Results:**

The procedure was completed in 82 minutes without placement of additional ports and without intraoperative complications. Estimated blood loss was 75 mL. The patient was discharged home the same day (4.3 hours postoperatively). Foley catheter removal on postoperative day 6 was followed by immediate urinary continence. Erectile function remained satisfactory at 3 months, indicating preservation of baseline functional outcomes. Final pathology demonstrated pT3b ISUP Grade Group 2 prostate cancer with evidence of prior ablation and negative surgical margins. At the most recent follow-up (12 months), PSA remained undetectable, with no biochemical recurrence.

**Conclusion:**

Transvesical SP-RARP appears to be a safe and effective salvage option following focal ablative therapy for prostate cancer. Leveraging the advantages of single-port robotic technology (8,9), this approach may facilitate outpatient surgery while maintaining favorable functional and oncologic outcomes.

**Available at:** VIDEO

## Data Availability

Uninformed
